# Long non-coding RNA UFC1 promotes gastric cancer progression by regulating miR-498/Lin28b

**DOI:** 10.1186/s13046-018-0803-6

**Published:** 2018-07-03

**Authors:** Xu Zhang, Wei Liang, Jibin Liu, Xueyan Zang, Jianmei Gu, Lei Pan, Hui Shi, Min Fu, Zhenhua Huang, Yu Zhang, Hui Qian, Pengcheng Jiang, Wenrong Xu

**Affiliations:** 1 Jiangsu Key Laboratory of Medical Science and Laboratory Medicine, School of Medicine, Jiangsu University, 301 Xuefu Road, Zhenjiang, 212013 Jiangsu China; 2 Institute of Digestive Diseases of Jiangsu University, The Affiliated People’s Hospital of Jiangsu University, 8 Dianli Road, Zhenjiang, 212002 Jiangsu China; 3 Tumor Institute, Nantong Tumor Hospital, 30 Tongyang North Road, Nantong, 226361 Jiangsu China

**Keywords:** UFC1, Lin28b, miR-498, Gastric cancer, Progression, Biomarker

## Abstract

**Background:**

Long non-coding RNAs (lncRNAs) have emerged as important regulators of human cancers. However, the functional roles of lncRNAs and the mechanisms responsible for their aberrant expression in gastric cancer (GC) have not been well characterized.

**Methods:**

In this study, we examined the expression of lncRNA UFC1 in GC by qRT-PCR and explored its correlation with clinicopathological parameters. In vitro cell functional assays and in vivo animal studies were performed to determine the roles of UFC1 in GC progression.

**Results:**

UFC1 was elevated and predicted poorer prognosis in GC. UFC1 knockdown inhibited while UFC1 overexpression promoted GC cell proliferation, migration, and invasion. UFC1 bound to miR-498 to antagonize its tumor suppressive effect on Lin28b. Suppression of Lin28b by miR-498 could be rescued by UFC1 overexpression, whereas Lin28b overexpression partially rescued UFC1 knockdown-mediated inhibition of GC cell function. Lin28b expression was increased in GC and suggested a co-expression pattern with UFC1.

**Conclusions:**

UFC1 has a promoting role in GC progression, at least in part, by acting as a miR-498 sponge and derepressing Lin28b expression, which would provide a novel biomarker for GC diagnosis and prognosis and offer a potential target for GC therapy.

**Electronic supplementary material:**

The online version of this article (10.1186/s13046-018-0803-6) contains supplementary material, which is available to authorized users.

## Background

Gastric cancer (GC) is one of the most common cancers and the third leading cause of cancer-related deaths worldwide. Most GC patients are diagnosed at advanced stages due to a lack of typical early symptoms. Though great efforts have been made to understand the molecular mechanisms for GC development and progression, yet it is still a great challenge to identify novel targets for GC detection and treatment [1].

Long non-coding RNAs (lncRNAs) have emerged as new players in human health and diseases. The alteration in lncRNA expression is found in various pathologic conditions including cancer [2, 3]. Increasing evidence suggests that lncRNAs are involved in cancer initiation, growth, metastasis, and therapy resistance [4]. Better understanding of the roles of lncRNAs in cancer may provide new biomarkers for early diagnosis and prognostic evaluation. In recent years, a series of lncRNAs have been reported in GC, either for their essential roles in GC progression or for their potential as diagnostic and prognostic biomarkers. For instance, KRT7-AS expression is upregulated in GC and it forms a duplex RNA-RNA structure with the host gene KRT7 mRNA to protect it from degradation, thereby promoting GC cell proliferation and migration [5]. PVT1 directly binds to FOXM1 protein to increase its stability, enhancing gastric cancer cell proliferation and invasion [6]. PVT1 is upregulated in gastric cancer and high level of PVT1 predicts poor prognosis in GC patients. GClnc1 is upregulated and associated with tumor size, metastasis, and poor prognosis in gastric cancer. GClnc1 acts as a modular scaffold for WDR5 (WD repeat domain 5) and KAT2A (Lysine Acetyltransferase 2A) to specify the histone modification pattern, thus promoting gastric cancer cell proliferation and invasiveness [7]. We have recently reported that ZFAS1 is upregulated in gastric cancer and its expression level was associated with GC progression [8]. Altogether, these findings suggest that lncRNA is critically involved in the pathogenesis of GC and may be utilized as biomarkers for GC diagnosis and prognosis.

Emerging evidence suggests that lncRNA can act as competitive endogenous RNA (ceRNA) to block miRNA-mediated target gene silencing [9, 10]. In gastric cancer, GAPLINC promotes the invasion of gastric cancer cells by binding to miR-211–3p, which upregulates the expression of CD44 [11]. BC032469 binds to miR-1207-5p to upregulate hTERT expression, promoting gastric cancer cell proliferation [12]. Moreover, SNHG5 interacts with miR-32 to promote gastric cancer cell proliferation and migration by targeting KLF4 [13]. These studies suggest that lncRNAs can participate in gastric cancer development and progression through the ceRNA mechanism.

UFC1 was first identified in hepatocellular carcinoma (HCC) as a target of miRNA-34a [14]. UFC1 binds to HuR, an mRNA stabilizing protein, to promote the expression of β-catenin. Yu et al. reported an increased UFC1 expression in colorectal cancer. Knockdown of UFC1 suppresses colorectal cancer cell proliferation and induces apoptosis though the activation of p38 signaling pathway [15]. However, the expression, clinical value, and biological roles of UFC1 in gastric cancer remain unclear.

In this study, we assessed UFC1 expression in GC and performed functional studies to explore the effects of UFC1 on GC progression. We demonstrated that UFC1 expression was upregulated in GC tissues, serum, and serum exosomes. The high level of UFC1 was associated with disease progression and predicted poor prognosis in GC patients. UFC1 knockdown inhibited while UFC1 overexpression promoted gastric cancer cell proliferation, migration and invasion. We revealed that UFC1 exerted its oncogenic activities by sponging miR-498 and acting as a ceRNA for Lin28b. Our findings suggest a promoting role of UFC1 in GC progression and provide a potential biomarker for GC diagnosis and prognosis.

## Methods

### Patients and tissue samples

A total of 79 paired gastric cancer and adjacent non-cancerous tissues (5 cm away from the tumor edge), 60 serum samples from GC patients, 35 serum samples from gastritis patients, and 40 serum samples from healthy donors were obtained from Department of General Surgery, the Affiliated People’s Hospital of Jiangsu University between April 2015 and September 2016. Written informed consent were obtained from all the patients and this study was approved by the Institutional Ethical Committee of Jiangsu University. All of the tissues were frozen in liquid nitrogen and then stored at − 80 °C for further use. The intravenous blood was centrifuged at 3000 g for 10 min and the serum were stored at − 80 °C until RNA extraction. The patients included in this study had not received any preoperative therapies.

### Cell culture

Human GC cell lines MGC-803, BGC-823, SGC-7901, HGC-27, and MKN-45 were purchased from the Cell Bank of the Chinese Academy of Sciences (Shanghai, China). Human normal gastric mucosa epithelial cell line GES-1 was obtained from Gefan Biological Technology (Shanghai, China). MGC-803 and HGC-27 cells were cultured in high glucose-DMEM with 10% fetal bovine serum (FBS; Invitrogen, Shanghai, China). GES-1, BGC-823, SGC-7901, and MKN-45cells were cultured in RPMI 1640 medium (Invitrogen) containing 10% FBS. All the cells were cultured in a 37 °C incubator with 5% CO_2_ atmosphere.

### RNA extraction and quantitative real time PCR

Total RNA was isolated from tissue samples and cells by using Trizol reagent (Invitrogen) according to the manufacturer’s procedures. Total RNA in serum was purified using miRNeasy Serum/Plasma kit according to the manufacturer’s instructions (Qiagen, Shanghai, China). The reverse transcription (RT) for mRNA, miRNA and lncRNA was carried out by using the miScript II RT Kit (Qiagen). Quantitative real time polymerase chain reaction was conducted with UltraSYBR Mixture (Cwbio, Beijing, China) on a real time PCR Detection System (CFX96, Bio-Rad, Shanghai, China). The target genes were normalized to U6 to obtain the relative expression level. The sequences of primers were provided in Additional file 1: Table S1.

### Western blot

Cells were lysed with RIPA buffer (Beyotime, Shanghai, China) containing protease inhibitors (Roche, CA, USA). Equal amounts of proteins were separated by SDS-polyacrylamide gel electrophoresis (SDS-PAGE) on a 12% polyacrylamide gel. The proteins were transferred electrophoretically onto 0.22 μm PVDF membranes (Millipore), blocked in 5% non-fat milk, and then incubated with primary antibodies against cyclin D1, Bcl2, Bax, Slug, Twist, Snail, E-cadherin, N-cadherin, and Vimentin (Cell Signaling Technology, Shanghai, China). After incubation with HRP-linked secondary antibody, the protein bands were visualized by using chemiluminescence (Millipore, Shanghai, China). GAPDH was used as the loading control.

### Gene overexpression and silencing

Cells were seeded in 6-well plates at a density of 2 × 10^5^/well and cultured in 37 °C incubator overnight. The overexpressing plasmid and knockdown shRNA (Hanbio, Shanghai, China) were transfected into the cells by using LipoFiter transfection reagent (Hanbio) in serum-free medium. Cells were changed to complete medium at 6 h after transfection and cultured for another 30 h. The target sequences of shRNAs were provided in Additional file 1: Table S2.

### Luciferase reporter assay

MGC-803 cells were co-transfected with miR-498 mimics (or miR-498 antago) and the luciferase reporter vector containing wild type (WT) or mutant (MUT) 3’-UTR of UFC1 (or Lin28b) as indicated. At 36 h after transfection, the cells were lysed and the luciferase activity was detected by using the dual luciferase assay kit (Promega, Madison, WI, USA).

### RNA immunoprecipitation assay

RNA immunoprecipitation (RIP) assay was performed by using a Magna RIP kit (Millipore) according to the instruction of the manufacturer. Whole cell lysate was incubated with RIP buffer containing magnetic beads which had been conjugated with human anti-Ago2 antibody, or normal mouse IgG as negative control. The immunoprecipitated RNAs were extracted, purified, and analyzed by using qRT- PCR to detect the binding of target RNAs.

### Cell cycle analysis

Cell cycle analysis was conducted with a cell cycle detection kit (Fcmacs, Jiangsu, China). The transfected cells were collected and fixed in 95% ethanol overnight. Afterwards, the cells were stained with 50 μg/ml propidium iodide (PI) for 30 min in dark. The cell cycle distribution was analyzed on a flow cytometer (BD, FACS Calibur) by using CellQuest software.

### Cell apoptosis assay

The Annexin V-Alexa Fluor 647/PI apoptosis detection kit (Fcmacs, Jiangsu, China) was used to detect cell apoptosis. After transfection for 24 h, the cells were digested with collagenase. The cells were collected and resuspended in binding buffer. Subsequently, the cells were stained with Annexin V-Alexa Fluor 647 and PI and then incubated for 15 min at room temperature. The apoptotic rate was analyzed by using flow cytometry.

### Cell counting assay and cell colony formation assay

The transfected cells were seeded in 24-well plates (1 × 10^4^ per well) and were counted for 6 days. For cell colony formation assay, the transfected cells were seeded in 6-well plates (1 × 10^3^/well) and cultured for 10 days. The medium was changed every 3 days. The cells were fixed with 4% paraformaldehyde and stained with crystal violet. The number of colonies was calculated under a microscope.

### Transwell migration assay

Cell migration assay were carried out by using transwell chambers with inserts of 8-μm pore size (Chemicon, Temecula, CA, USA). A total of 2 × 10^4^ cells were seeded into the top chamber in 200 μL serum-free media and 600 μL complete medium was added into the bottom chamber. After 24 h, the cells were fixed with 4% paraformaldehyde and stained with crystal violet. The number of migrated cells was counted from five randomly selected field and then averaged.

### Matrigel invasion assay

After matrigel (BD Biosciences, Shanghai, China) was added on the transwell chamber and clotted, a total of 1 × 10^5^ cells were seeded into the top chamber in 200 μL serum-free media. The bottom well was added with 600 μL complete medium and cells were allowed to invade for 36 h. The matrigel and the cells on the top chamber were removed with cotton swab. The cells invaded through the pore were fixed with 4% paraformaldehyde and stained with crystal violet. The number of invaded cells were counted from five randomly selected fields and averaged.

### In vivo animal studies

BALB/c nude mice aged 4–6 weeks were purchased from the Slac Laboratory Animal Center (Shanghai, China) and maintained in accordance with the institutional policies. Sh-UFC1 or sh-control stably transfected cells were collected in PBS and subcutaneously injected into the mice (2 × 10^6^ cells/mice, *n* = 6). Tumor size was assessed every 3 days and tumor volumes were calculated using the formula: V = 0.5 × D × d^2^ where V represents volume, D represents longitudinal diameter and d represents latitudinal diameter. The protocol was approved by the Animal Use and Care Committee of Jiangsu University.

### Immunohistochemistry

For immunohistochemical analyses, 4% paraformaldehyde fixed tissues were embedded in paraffin and cut into 4 μm-thick sections. The sections were incubated with primary monoclonal antibody against Ki-67 (Cell Signaling Technology) followed by incubation with the secondary antibody for 30 min at room temperature. After being incubated with 3, 3′-Diaminobenzidine (3, 3’-DAB, Maxim, Fuzhou, China) for 5 min, the sections were counterstained with hematoxylin for 30 s. Finally, the sections were photographed under a TE2000 microscope (Nikon, Tokyo, Japan).

### Statistical analysis

Statistical analyses were carried out by using the SPSS 22.0 software (Chicago, IL, USA). All the experiments were performed for at least three times, and all values presented as mean values ± SD. The Student’s t test was used for comparisons between paired groups. The Pearson χ2 test was used for the associations between UFC1 and the clinicopathological features. The diagnostic value of UFC1 was evaluated by receiver operating characteristic (ROC) curve. Survival time was analyzed by Kaplan–Meier method and log-rank test. *P* values less than 0.05 was considered statistically significant.

## Results

### UFC1 is highly expressed in gastric cancer and high level of UFC1 predicts poor prognosis

**Fig. 1:**
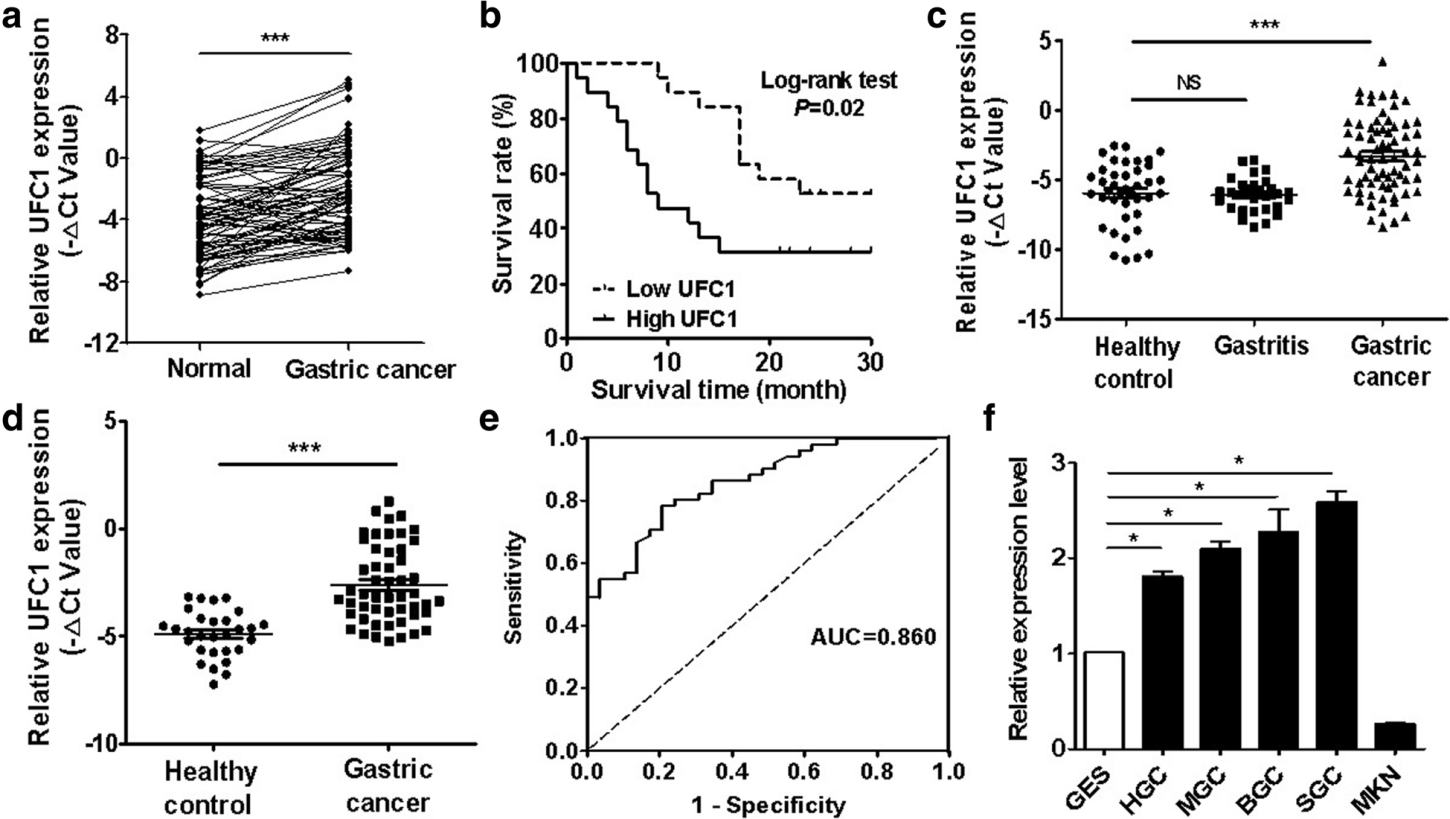
UFC1 is upregulated in gastric cancer and its increased expression predicts poor prognosis. **a** QRT-PCR analyses of UFC1 expression in 79 paired gastric cancer GC tissues and matched adjacent normal tissues normal. **b** The prognostic value of UFC1 expression level in gastric cancer. **c** QRT-PCR analyses of UFC1 expression in the serum of patients with gastric cancer (*n* = 60), gastritis (*n* = 35), and healthy controls (*n* = 40). **d** QRT-PCR analyses of UFC1 expression in the exosomes from the serum of gastric cancer patients (*n* = 57) and healthy controls (*n* = 29). **e** ROC curve for the diagnostic value of UFC1 in the serum exosomes of gastric cancer patients. **f** QRT-PCR analyses of UFC1 expression in human GC cell lines and normal gastric mucosa epithelial cell line. *P < 0.05; ***P < 0.001

We first detected the relative expression levels of UFC1 in 79 paired gastric cancer tissues and adjacent non-tumor tissues. The results showed that 64.6% (51/79) of GC tissues exhibited at least two-fold increase in UFC1 expression level compared to the paired non-cancerous tissues (Fig. 1a, *P* < 0.001). We then explored the correlation between UFC1 expression level and clinicopathological parameters. The expression level of UFC1 was positively correlated with tumor size, TNM stage and lymphatic metastasis (Additional file 1: Table S3). Furthermore, the patients who had high levels of UFC1 came out with a notably poorer prognosis than those who had low levels of UFC1 (Fig. 1b, *P* < 0.05).We next examined the expression level of UFC1 in the serum of patients with gastric cancer. The serum levels of UFC1 were elevated in GC patients compared to that in gastritis patients and healthy controls (Fig. 1c, *P* < 0.001). We then determined the correlation between the expression levels of UFC1 in serum and the clinicpathological parameters. The results of correlation analyses showed that the serum levels of UFC1 were positively associated with TNM stage and lymphatic metastasis (Additional file 1: Table S4). We further isolated exosomes from the serum samples and detected the expression levels of UFC1 in exosomes. We found that the expression level of exosomal UFC1 was increased in GC patients compared to that in healthy controls (Fig. 1d). The levels of exosomal UFC1 were also positively associated with TNM stage and lymphatic metastasis (Additional file 1: Table S5). The area under the receiver operating characteristic (ROC) curve for exosomal UFC1 was 0.860 (95% CI, 0.780 to 0.900, Fig. 1e). Finally, we assessed the expression levels of UFC1 in GC cell lines and normal gastric mucosa epithelial cell line. The results showed that UFC1 expression was higher in GC cell lines (including HGC-27, MGC-803, BGC-823 and SGC-7901) than that in normal gastric mucosa epithelial cell line GES-1 (Fig. 1f).

### UFC1 knockdown inhibits while UFC1 overexpression promotes gastric cancer cell proliferation, migration and invasion

**Fig. 2:**
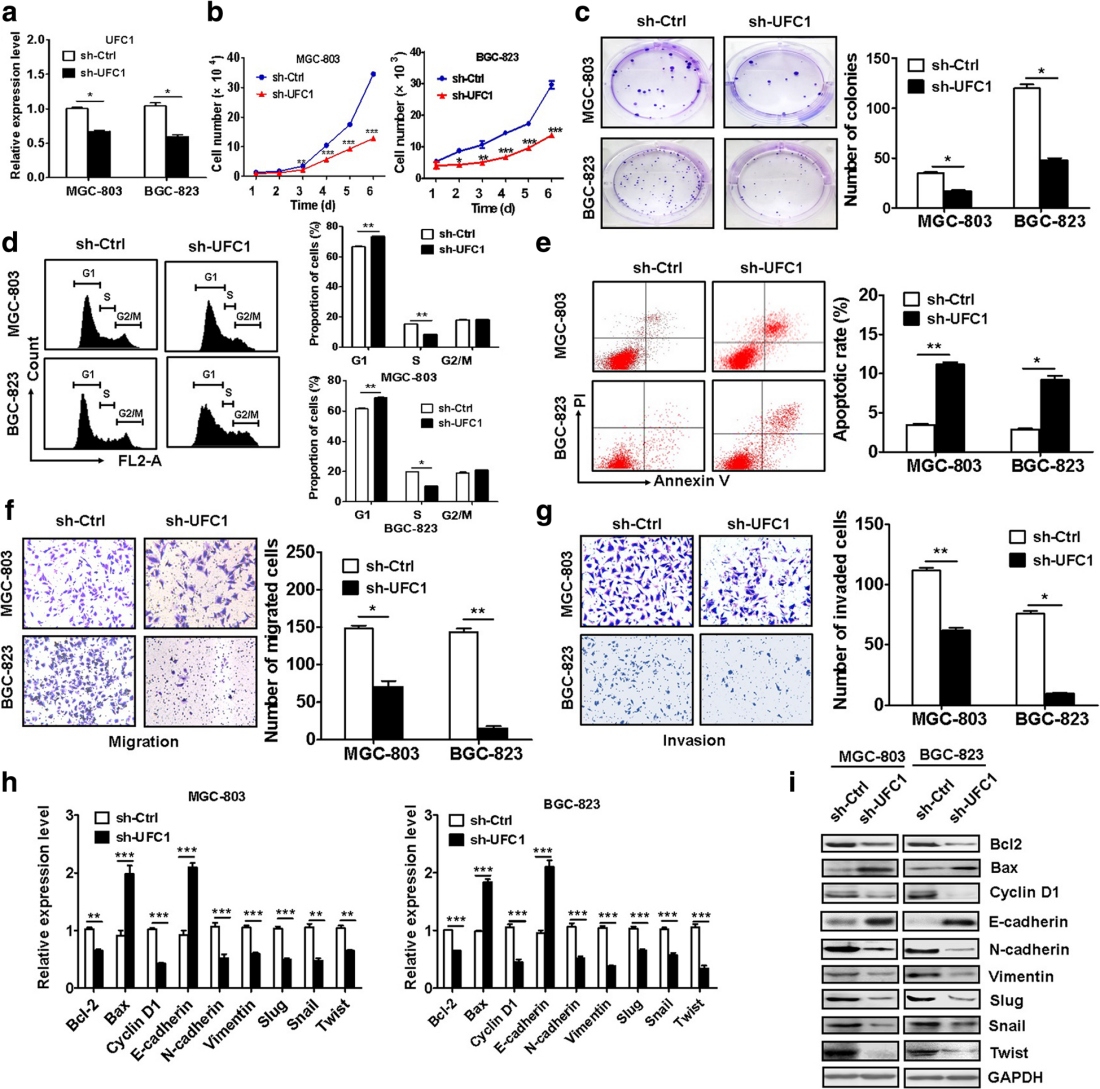
UFC1 knockdown inhibits gastric cancer cell proliferation, migration and invasion. **a** UFC1 expression was silenced in gastric cancer cells by using shRNA. The efficiency of gene knockdown was validated. **b** The growth of sh-UFC1 transfected GC cells was determined by using cell counting assay. **c** Cell colony formation assays for the proliferating ability of sh-UFC1 transfected GC cells. **d** The cell cycle distribution in sh-UFC1 transfected GC cells was determined by using flow cytometry. **e** Flow cytometric analyses of cell apoptosis in sh-UFC1 transfected GC cells. **f** The effects of UFC1 knockdown on the migration of GC cells were determined by using transwell migration assay. **g** Matrigel invasion assay was performed to determine the effects of UFC1 knockdown on GC cell invasion. **h** The effects of UFC1 knockdown on the expression of growth and metastasis-related genes in GC cells. **i** The effects of UFC1 knockdown on the expression of growth and metastasis-related proteins in GC cells

The increased expression of UFC1 in GC led us to hypothesize that UFC1 might function as an oncogene in GC. To this end, we knocked down UFC1 expression in GC cells by using shRNA. The knockdown efficiency was verified by using qRT-PCR (Fig. 2a). The results of cell counting assay showed that UFC1 knockdown inhibited gastric cancer cell proliferation (Fig. 2b), which was further confirmed by the results of colony formation assays (Fig. 2c). GC cells transfected with UFC1 shRNA had decreased S phase and increased apoptosis (Fig. 2d, e). The number of migrated and invaded cells was decreased in sh-UFC1 group compared to that in control group (Fig. 2f and g). The inhibitory effects of UFC1 knockdown on GC cell proliferation, migration and invasion were further confirmed by using another UFC1-targeting shRNA (Additional file 2: Figure S1). Moreover, the expression levels of Bcl-2 and cyclin D1 was decreased in shRNA transfected cells (Fig. 2h and i). The expression of epithelial marker E-cadherin was increased while that of mesenchymal marker N-cadherin and Vimentin was decreased in UFC1 knockdown cells. In addition, the expression of EMT transcription factors including Slug, Snail, and Twist was significantly downregulated in sh-UFC1 transfected cells (Fig. 2h, i). In contrast to that observed for UFC1 knockdown, UFC1 overexpression promoted GC cell proliferation, migration and invasion (Additional file 2: Figure S2).

### UFC1 functions as a miR-498 sponge in gastric cancer cells

**Fig. 3:**
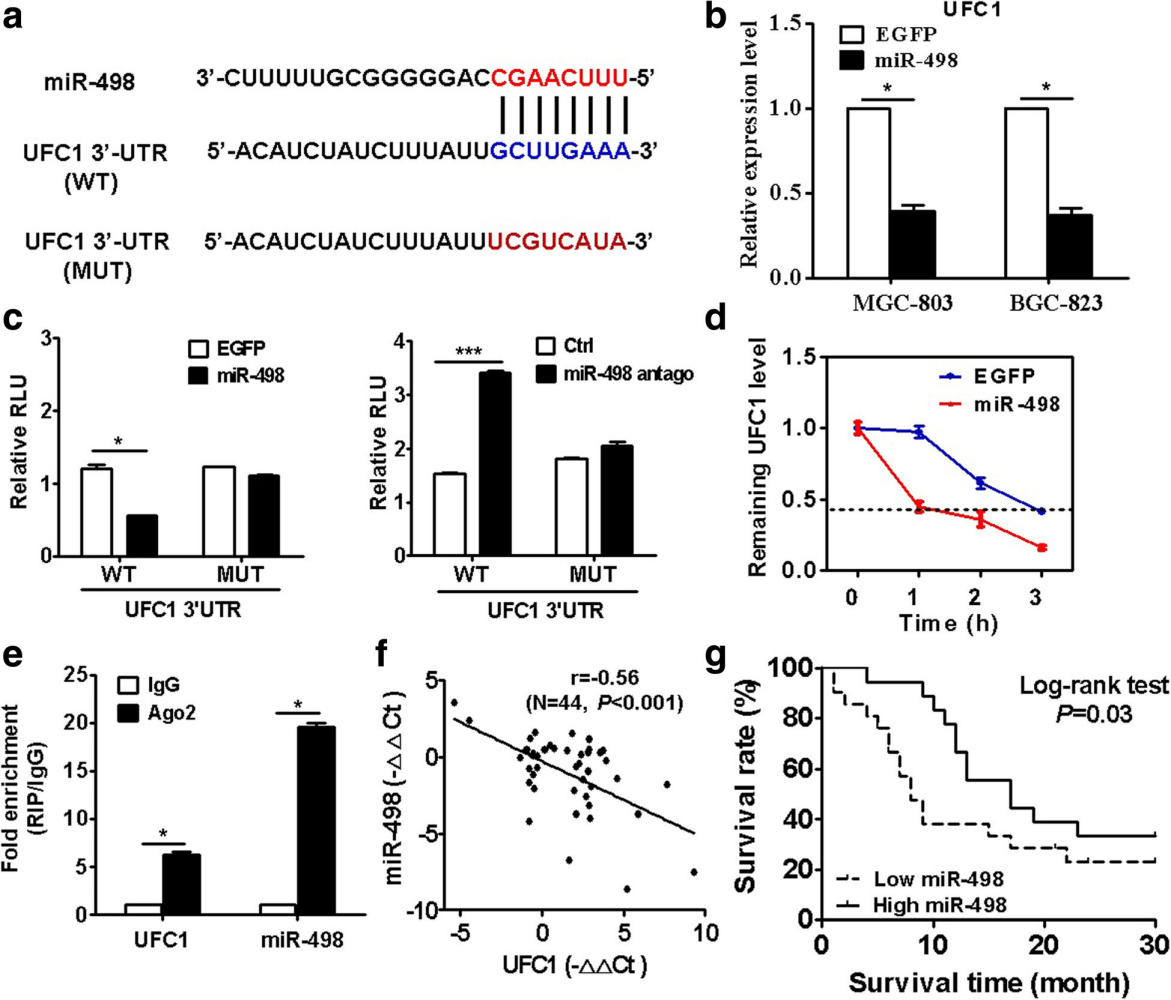
UFC1 interacts with miR-498 in gastric cancer cells. **a** The potential binding site of miR-498 in UFC1 was predicted by using bioinformatic analyses. **b** QRT-PCR analyses of UFC1 expression in GC cells transfected with miR-498. **c** GC cells were co-transfected with UFC1 3’-UTR reporter plasmid wild type or mutant with miR-498 or miR-498 antago. The luciferase activity was determined by using dual-luciferase reporter assay. **d** GC cells transfected with vector EGFP or miR-498 were treated with actinomycin D for different times. The relative expression levels of UFC1 were determined by using qRT-PCR. **e** RIP assay for the binding of UFC1 with Ago2 protein. **f** The expression of miR-498 in gastric cancer tissues was examined by using qRT-PCR. The association between miR-498 and UFC1 expression level was determined by using Pearson correlation analysis. **g** The prognostic values of miR-498 expression level in gastric cancer

LncRNAs could function as ceRNAs to regulate target gene expression by binding to miRNA. We predicted the potential targets of UFC1 by searching the bioinformatic database miRDB and identified miR-498 as a candidate binding miRNA (Fig. 3a and Additional file 2: Figure S3). Thus, we attempted to analyze the regulation of UFC1 by miR-498. QRT-PCR results showed that UFC1 expression was downregulated in miR-498 transfected GC cells (Fig. 3b). Luciferase reporter assay results showed that the luciferase activity of UFC1–3’UTR was significantly decreased in miR-498 transfected group. On the contrary, the luciferase activity of UFC1–3’UTR was increased in miR-498 antago transfected cells (Fig. 3c). However, no significant difference was found in the luciferase activities of mutant UFC1–3’UTR between miR-498 (or miR-498 antago) group and control group (Fig. 3c). In addition, the half-life of UFC1 was remarkably shortened in GC cells transfected with miR-498 (Fig. 3d). RIP assay results showed that UFC1 and miR-498 were co-immunoprecipitated by the anti-Ago2 antibody but not the IgG antibody (Fig. 3e). The expression level of miR-498 was significantly lower in tumor tissues than that in adjacent non-tumor tissues (Additional file 2: Figure S4A). In support of this finding, miR-498 expression level was lower in gastric cancer cell lines than that in normal gastric mucosa epithelial cell line (Additional file 2: Figure S4B). Moreover, there was a negative association between the expression level of UFC1 and that of miR-498 in tumor tissues (*r* = − 0.52, *P* < 0.001, Fig. 3f). GC patients with high levels of miR-498 had a better prognosis that those with low levels of miR-498 (Fig. 3g, *P* < 0.05). Taken together, these findings suggest that UFC1 function as a miR-498 sponge in gastric cancer cells.

### MiR-498 overexpression inhibits gastric cancer cell proliferation, migration and invasion

**Fig. 4:**
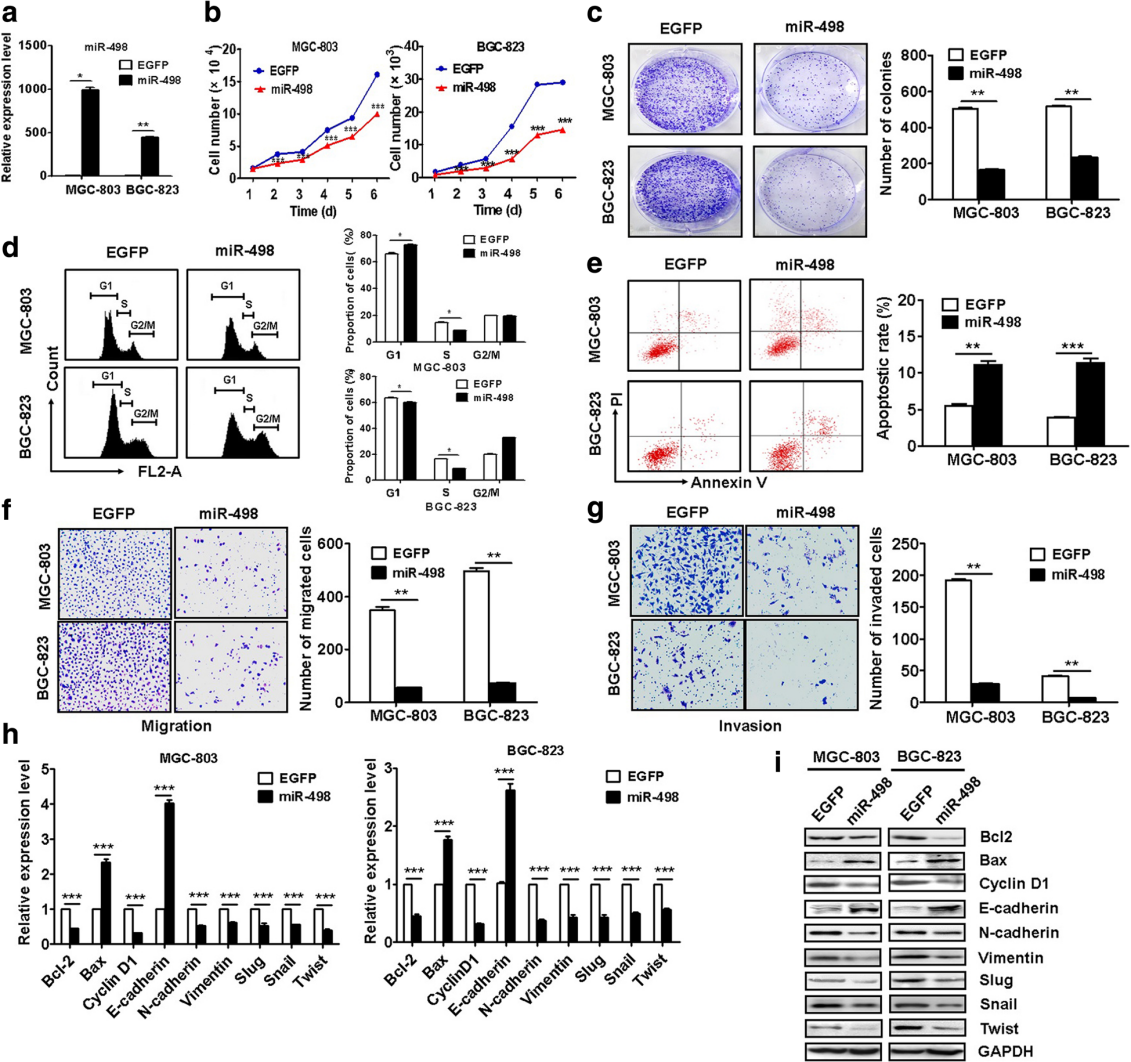
MiR-498 inhibits gastric cancer proliferation, migration and invasion. **a** The efficiency for miR-498 transfection in GC cells was verified by qRT-PCR. **b** The growth of miR-498 overexpressing GC cells was determined by using cell counting assay. **c** Cell colony formation assays for the proliferating ability of miR-498 overexpressing GC cells. **d** The cell cycle distribution in miR-498 overexpressing GC cells was determined by using flow cytometry. **e** Flow cytometric analyses of cell apoptosis in miR-498 overexpressing GC cells. **f** The effects of miR-498 overexpression on the migration of GC cells were determined by using transwell migration assay. **g** Matrigel invasion assay was performed to determine the effects of miR-498 overexpression on GC cell invasion. **h** The effects of miR-498 overexpression on the expression of growth and metastasis-related genes. **i** The effects of miR-498 overexpression on the expression of growth and metastasis-related proteins in GC cells

We next determined the biological roles of miR-498 in GC. The results of cell counting and colony formation assays showed that miR-498 overexpression inhibited GC cell proliferation (Fig. 4a, b, c). MiR-498 overexpression increased the percentage of cells at G1 phase while decreased that of cells at S phase (Fig. 4d). The rate of apoptotic cells increased when miR-498 was overexpressed in GC cells (Fig. 4e). In addition, miR-498 overexpression inhibited the migration and invasion of GC cells (Fig. 4f, g). In consistent with that observed for UFC1 knockdown, miR-498 overexpression also led to the downregulation of Bcl-2, cyclin D1 and EMT transcription factors including Slug, Snail, and Twist in GC cells (Fig. 4h, i). However, UFC1 co-transfection reversed the suppressive roles of miR-498 in GC cell proliferation, migration and invasion (Additional file 2: Figure S5).Taken together, these findings suggest that miR-498 plays a tumor suppressive role in gastric cancer.

### Lin28b is a downstream target of miR-498

**Fig. 5:**
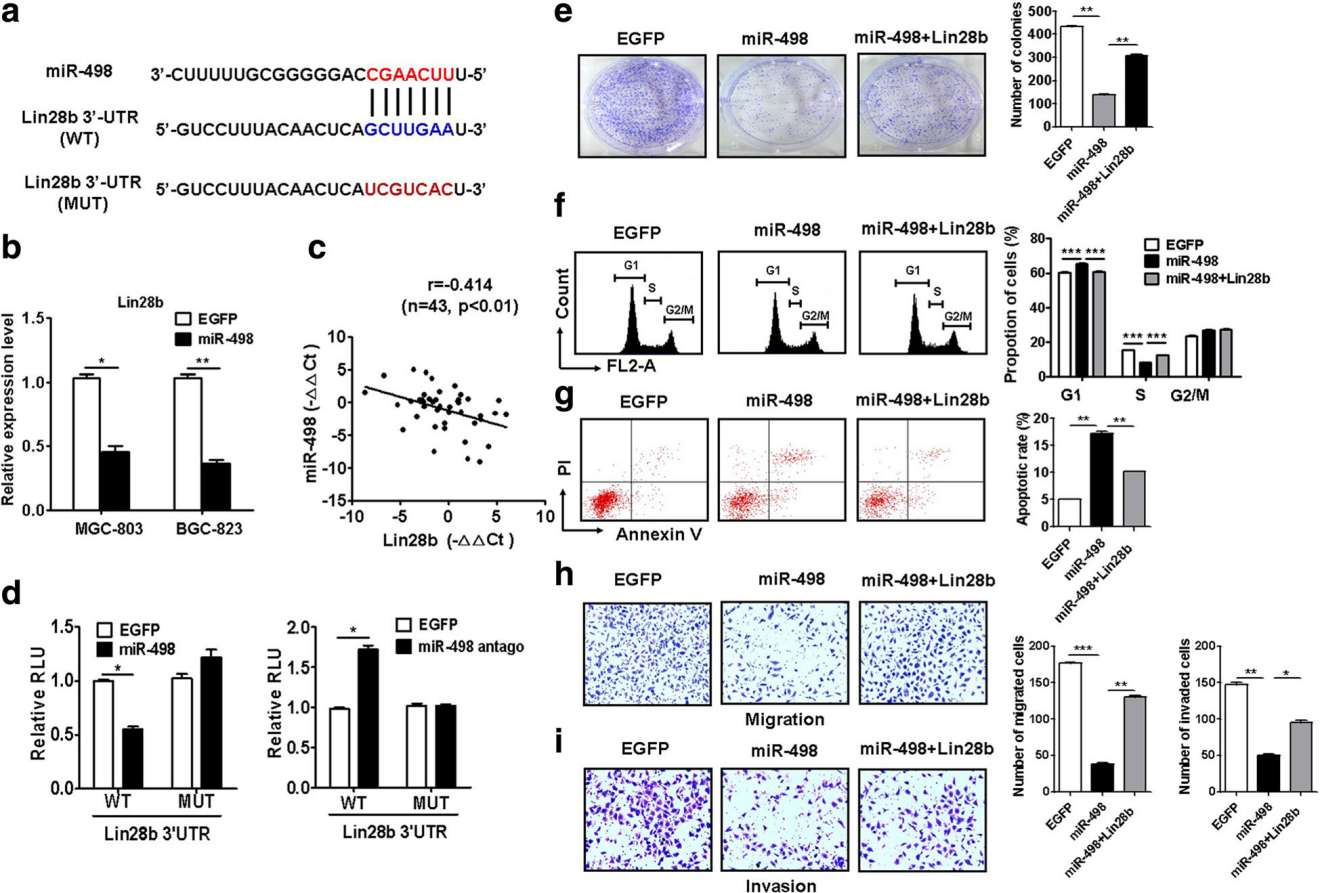
Lin28b is a downstream target ofmiR-498. **a** The potential binding site in Lin28b for miR-498 was predicted by using bioinformatic analyses. **b** QRT-PCR analyses of Lin28b expression in GC cells transfected with miR-498. **c** The expression of Lin28b in gastric cancer tissues was examined by using qRT-PCR. The association between Lin28b and miR-498 expression levels was determined by using Pearson correlation analysis. **d** GC cells were co-transfected with Lin28b 3’-UTR reporter plasmid wild type or mutant with miR-498 plasmid or miR-498 antago. The luciferase activity was determined by using dual-luciferase reporter assay. **e** GC cells were transfected with miR-498 in the presence or absence of Lin28b. The proliferating ability of GC cells was determined by using cell colony formation assay. **f** Cell cycle distribution was determined by using flow cytometry. **g** Cell apoptosis was determined by using flow cytometry. **h** The migration ability of GC cells were determined by using transwell migration assay. **i** The invasion ability of GC cells were determined by using matrigel invasion assay

We then predicted the potential target genes of miR-498 by using online miRNA target prediction tools including Targetscan, DIANA, and miRDB and identified Lin28b as a potential downstream gene of miR-498 (Fig. 5a and Additional file 2: Figure S3). Lin28b expression was downregulated in miR-498 transfected GC cells (Fig. 5b). There was a negative association between the expression level of Lin28b and that of miR-498 in tumor tissues (*r* = − 0.414, *P* < 0.01, Fig. 5c). Moreover, the expression level of Lin28b was significantly upregulated in gastric cancer tissues and gastric cancer cell lines compared to non-tumor tissues and normal gastric mucosa epithelial cell line, respectively (Additional file 2: Figure S4C, D). MiR-498 overexpression decreased while miR-498 antago increased the luciferase activity of wild type Lin28b-3’UTR (Fig. 5d). However, no significant difference was found in the luciferase activity of mutant Lin28b-3’UTR between miR-498 (or miR-98 antago) and control groups.

We further explored the biological roles of Lin28b in GC. We found that Lin28b knockdown inhibited GC cell proliferation (Additional file 2: Figure S6A, B, C). Lin28b knockdown induced cell cycle arrest and apoptosis in GC cells (Additional file 2: Figure S6D, E). Lin28b knockdown also inhibited the migration and invasion of GC cells (Additional file 2: Figure S6F, G). The expression of Bcl-2, Cyclin D1, and EMT transcription factors including Slug, Snail, and Twist was downregulated in Lin28b knockdown GC cells (Additional file 2: Figure S6H, I). On the contrary, Lin28b overexpression led to the opposite effects (Additional file 2: Figure S7).

We conducted rescue experiments to determine the importance of Lin28b in miR-498-regulated tumor suppression in GC. The co-transfection of Lin28b rescued the inhibitory roles of miR-498 in the proliferation, migration and invasion of GC cells (Fig. 5e, h, i). Lin28b co-transfection also reversed the inducing effects of miR-498 on cell cycle arrest and apoptosis in GC cells (Fig. 5f, g). Therefore, these data suggest that Lin28b is an important downstream target of miR-498.

### UFC1 promotes gastric cancer growth in vivo through the regulation of Lin28b

**Fig. 6:**
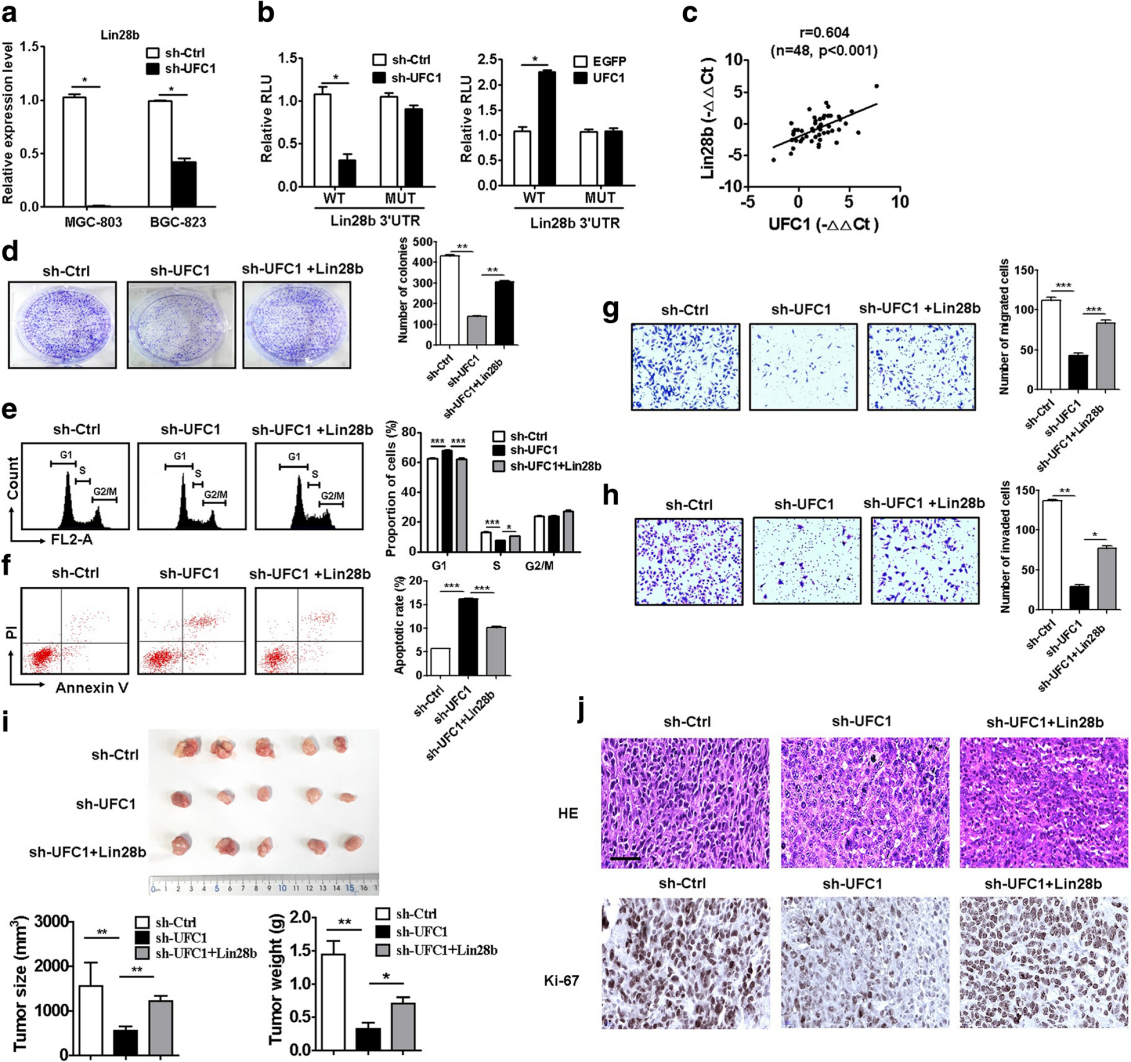
UFC1 promotes gastric cancer growth through the regulation of Lin28b. **a** RT-PCR analyses of Lin28b expression in control and UFC1 knockdown GC cells. **b** GC cells were co-transfected with Lin28b 3’-UTR reporter plasmid wild type or mutant with sh-UFC1 or UFC1 plasmid. The luciferase activity was determined by using dual-luciferase reporter assay. **c** The association between Lin28b and UFC1 expression levels was determined by using Pearson correlation analysis. **d** GC cells were transfected with sh-UFC1 in the presence or absence of Lin28b. The proliferating ability of GC cells was determined by using cell colony formation assay. **e** Cell cycle distribution was determined by using flow cytometry. **f** Cell apoptosis was determined by using flow cytometry. **g** The migration ability of GC cells were determined by using transwell migration assay. **h** The invasion ability of GC cells were determined by using matrigel invasion assay. **i** Tumor growth curves and tumor weights for mice in sh-control, sh-UFC1, and sh-UFC1 + Lin28b groups. **j** HE staining and immunohistochemical staining of Ki-67 for tumor tissues from mice in sh-control, sh-UFC1, and sh-UFC1 + Lin28b groups. Scale bar: 100 μm

Lin28b expression was downregulated in UFC1 knockdown GC cells (Fig. 6a). The luciferase activity of Lin28b-3’UTR was increased in UFC1-overexpressing group but decreased in UFC1 knockdown group (Fig. 6b). There was no significant difference in the luciferase activity of mutant Lin28b-3’UTR between UFC1 (or sh-UFC1) and control groups (Fig. 6b). On the contrary, a positive association between the expression level of UFC1 and that of Lin28b was observed in gastric cancer tissues (*r* = 0.604, *P* < 0.001, Fig. 6c).

We conducted rescue experiments to determine the importance of Lin28b in UFC1-regulated tumor progression in GC. The co-transfection of Lin28b rescued the inhibitory roles of UFC1 knockdown in the proliferation, migration and invasion of GC cells (Fig. 6d, g, h). Lin28b co-transfection also reversed the inducing effects of UFC1 knockdown on cell cycle arrest and apoptosis in GC cells (Fig. 6e, f). On the contrary, the promoting roles of UFC1 overexpression in GC cell proliferation, migration and invasion were reversed by Lin28b knockdown (Additional file 2: Figure S8).

To further confirm the significance of Lin28b regulation by UFC1 in tumor growth, UFC1 knockdown cells co-transfected with or without Lin28b were subcutaneously injected into nude mice. The mean tumour size and tumor weight in sh-UFC1 group were remarkably lower than that in control group (Fig. 6i). However, the mean tumor size and tumor weight in Lin28b co-transfection group were remarkably higher than that in sh-UFC1 alone group (Fig. 6i). In addition, the results of immunohistochemical analyses showed that the tumors tissues from UFC1 knockdown group displayed weaker Ki-67 staining than those from the control group (Fig. 6j). However, the tumors tissues from Lin28b co-transfection group displayed stronger Ki-67 staining than those from sh-UFC1 alone group (Fig. 6j). Taken together, these findings suggest that UFC1 promotes GC growth through the upregulation of Lin28b.

## Discussion

In this study, we reported the increased expression of lncRNA UFC1 in tumor tissues, serum and serum exosomes of GC patients. We revealed that UFC1 upregulation was closely associated with disease progression and poor prognosis in GC patients. We demonstrated that UFC1 knockdown inhibited while UFC1 overexpression promoted gastric cancer cell proliferation, migration, and invasion. UFC1 exerted its oncogenic activities in gastric cancer by sponging miR-498 and derepressing its downstream target Lin28b. The identification of UFC1/miR-498/Lin28b signaling axis thus adds new evidence to the important roles of lncRNAs in gastric cancer progression and provides new targets for gastric cancer diagnosis, prognosis and therapy.

The circulating lncRNAs provide a blood-based biomarker for cancer detection [16]. We identified an increased expression of UFC1 in the serum of gastric cancer patients, which suggests that UFC1 may serve as a potential marker for monitoring progression and prognosis of GC. Moreover, UFC1 was present in the serum exosomes of gastric cancer patients. Exosomes have emerged as a new biomarker for liquid biopsy [17]. The high level of exosomal UFC1 could distinguish gastric cancer patients from healthy controls, indicating an important value of serum exosomal UFC1 in GC diagnosis.

UFC1 knockdown induced cell cycle arrest and cell apoptosis, leading to the inhibition of gastric cancer cell proliferation. UFC1 knockdown also retarded gastric cancer growth in vivo, indicating that UFC1 is critical for gastric carcinogenesis. In addition, UFC1 knockdown reversed EMT phenotype of GC cells and inhibited their migration and invasion, suggesting a key role of UFC1 in gastric cancer metastasis. Exosomes-mediated transfer of non-coding RNAs has been suggested as an important mechanism for cancer progression [18]. We have previously shown that exosomes could transfer lncRNA ZFAS1 to promote gastric cancer cell proliferation, migration and invasion [8]. In this study, we found that exosomes also contained UFC, suggesting that exosome-mediated transfer of oncogenic lncRNAs may represent a common mechanism for gastric cancer progression.

Recently, Song et al. have identified a regulatory network in gastric cancer whereby claudin-4 expression is reduced by miR-596 and miR-3620-3p, which are in turn bound by lncRNA-KRTAP5-AS1 and lncRNA-TUBB2A acting as ceRNAs, resulting in increased claudin-4 expression, suggesting that non-coding RNAs play important roles in the regulatory network of oncogenes and tumor suppressors in gastric cancer [19]. In this study, we identified UFC1 as a ceRNA by binding to miR-498. UFC1 expression was negatively associated with that of miR-498 in gastric cancer. Moreover, UFC1 overexpression antagonized the suppressive role of miR-498 in gastric cancer cell proliferation, migration and invasion. The reduced expression and tumor-suppressive role of miR-498 have been found in esophageal squamous cell carcinoma [20], colorectal cancer [21], non-small cell lung cancer [22], and ovarian cancer [23]. However, in triple negative breast cancer [24] and oral tongue squamous cell carcinoma [25], miR-498 has been shown to play promoting roles in cancer cell proliferation and invasion, suggesting that the functions of miR-498 may be cancer type-specific and cell context-dependent. In this study, we demonstrated that miR-498 expression was downregulated in gastric cancer and low level of miR-498 predicted poor prognosis for patients with gastric cancer. MiR-498 overexpression inhibited GC cell proliferation, migration and invasion. Thus, miR-498 may represent a new target for gastric cancer diagnosis and therapy.

The previous studies have shown that miR-498 could target FOXO3 (forkhead box O3) and hTERT (human telomerase reverse transcriptase) to inhibit cancer cell proliferation [26, 27]. In this study, we identified Lin28b as a new target of miR-498. Lin28b is an evolutionarily conserved RNA-binding protein implicated in maintaining the pluripotency of stem cells [28]. Overexpression of Lin28b has been observed in human cancers and Lin28B upregulation is associated with poor prognosis and tumor recurrence [29]. Lin28b acts as an oncogene and facilitates tumor progression though let-7-dependent and -independent mechanisms [30, 31]. Wang et al. demonstrate that the silencing of Lin28b inhibits cell proliferation and migration by inducing cell cycle arrest and suppressing EMT in pancreatic ductal adenocarcinoma cells [32]. We found that Lin28b expression was increased in gastric cancer tissues and was negatively associated with that of miR-498. Lin28b knockdown recapitulated the effects of miR-498 overexpression on gastric cancer cell proliferation, migration and invasion. On the contrary, Lin28b knockdown reversed the promoting role of UFC1 in gastric cancer progression, suggesting that the upregulation of Lin28b contributes, at least in part, to the oncogenic role of UFC1 in gastric cancer.

LncRNAs regulate gene expression through distinct mechanisms. Liu et al. demonstrate that HOXA11-AS could interact with WDR5 to promote β-catenin transcription, bind with EZH2 to repress p21 transcription, and induce KLF2 mRNA degradation via interacting with STAU1, thus promoting gastric cancer growth and metastasis [33]. Wang et al. suggest that UCA1 promotes Cbl-c-mediated GRK2 ubiquitination and degradation, activating ERK-MMP9 signaling pathway and increasing the metastatic ability of gastric cancer cells [34]. UFC1 has been previously shown to promote the expression of β-catenin by binding to its mRNA [14]. We reported here that UFC1 could sponge miRNA to regulate the expression of Lin28b. Whether UFC1 can perform oncogenic roles through the regulation of protein stability warrants further investigation.

## Conclusions

Collectively, we demonstrated that UFC1 was upregulated in tumor tissues, serum, and serum exosomes of GC patients. UFC1 promoted gastric cancer cell proliferation, migration and invasion by favoring cell cycle progression, inhibiting cell apoptosis and inducing EMT. UFC1 acted as a ceRNA for Lin28b oncogene by sponging tumor-suppressive miR-498. Our findings not only suggest a critical role of UFC1 in gastric cancer progression but also provide a novel biomarker for gastric cancer diagnosis and therapy.

## Additional files

Additional file 1: **Table S1.** The sequences of primers for qRT-PCR. **Table S2** The sequences of control and UFC1 shRNAs. **Table S3.** The association between UFC1 expression levels (–ΔCt) in tumor tissues and the clinicopathological features of gastric cancer patients. **Table S4.** The correlation between serum UFC1 expression levels (–ΔCt) and the clinicopathological characteristics of gastric cancer patients. **Table S5.** The correlation between exosomal UFC1 expression levels (–ΔCt) and the clinicopathological parameters of gastric cancer patients. (DOCX 25 kb)

Additional file 2: **Figure S1.** UFC1 knockdown inhibits gastric cancer cell proliferation, migration and invasion. **Figure S2.** UFC1 overexpression enhances gastric cancer cell proliferation, migration and invasion. **Figure S3.** Bioinformatic prediction of UFC1-binding miRNAs and target genes of miR-498. **Figure S4.** Relative expression levels of miR-498 and Lin-28b in gastric cancer cells and gastric cancer tissues. **Figure S5.** UFC1 overexpression antagonizes miR-498-medited inhibition of gastric cancer cell proliferation, migration and invasion. **Figure S6.** Lin28b knockdown inhibits gastric cancer cell proliferation, migration and invasion. **Figure S7.** Lin28b overexpression promotes gastric cancer cell proliferation, migration and invasion. **Figure S8.** UFC1 promotes gastric cancer cell proliferation, migration and invasion via the upregulation of Lin28b. (DOCX 19 kb)

## Abbreviations

ceRNA: Competitive endogenous RNA
EMT: Epithelial-mesenchymal transition
FOXM1: Forkhead box protein M1
GC: Gastric cancer
HCC: Hepatocellular carcinoma
hTERT: Human telomerase reverse transcriptase
KAT2A: Lysine acetyltransferase 2A
KLF4: Krüppel-like factor4
LncRNAs: Long non-coding RNAs
MMP9: Matrix metallopeptidase 9
RIP: RNA Immunoprecipitation
ROC: Receiver operating characteristic
WDR5: WD repeat domain 5
ZFAS1: ZNFX1 antisense RNA1
