# Supplementary material for: Long non-coding RNA UFC1 promotes gastric cancer progression by regulating miR-498/Lin28b
Source: J Exp Clin Cancer Res. 2018 Jul 3;37:134. doi: 10.1186/s13046-018-0803-6 (PMC6029056; doi:10.1186/s13046-018-0803-6)
Supplement: Supplementary file 1 — Table S1. The sequences of primers for qRT-PCR. Table S2 The sequences of control and UFC1 shRNAs. Table S3. The association between UFC1 expression levels (–ΔCt) in tumor tissues and the clinicopathological features of gastric cancer patients. Table S4. The correlation between serum UFC1 expression levels (–ΔCt) and the clinicopathological characteristics of gastric cancer patients. Table S5. The correlation between exosomal UFC1 expression levels (–ΔCt) and the clinicopathological parameters of gastric cancer patients. (DOCX 25 kb) [file 13046_2018_803_MOESM1_ESM.docx]

**Table S1** The sequences of primers for qRT-PCR

| Gene | Sequence | Product Size (bp) | Annealing Temperature(^o^C) |
| --- | --- | --- | --- |
| U6 | F:5’-CTCGCTTCGGCAGCACA-3’ | 94 | 55 |
|  | R:5’-AACGCTTCACGAATTTGCGT-3’ |  |  |
| UFC1 | F: 5’-TCCAACCTGAGTGACATAGCGA-3’ | 172 | 55 |
|  | R: 5’-CTGACCTCCAACTCCAACGAAT-3’ |  |  |
| E-cadherin | F:5’-CGCATTGCCACATACACTCT-3’ | 252 | 55 |
|  | R:5’-TTGGCTGAGGATGGTGTAAG-3’ |  |  |
| N-cadherin | F:5’-AGTCAACTGCAACCGTGTCT-3’ | 337 | 55 |
|  | R:5’-AGCGTTCCTGTTCCACTCAT-3’ |  |  |
| Slug | F:5’-CCTGGTTGCTTCAAGGACAC-3’ | 395 | 55 |
|  | R:5’-TCCATGCTCTTGCAGCTCTC-3’ |  |  |
| Snail | F:5’-GCGAGCTGCAGGACTCTAAT-3’ | 310 | 55 |
|  | R:5’-GCCTCCAAGGAAGAGACTGA-3’ |  |  |
| Twist | F:5’-ACGAGCTGGACTCCAAGATG-3’ | 484 | 55 |
|  | R:5’-GGCACGACCTCTTGAGAATG-3’ |  |  |
| Vimentin | F:5’-GAGCTGCAGGAGCTGAATG-3’ | 344 | 55 |
|  | R:5’- AGGTCAAGACGTGCCAGAG-3’ |  |  |
| Bax | F:5’-CACCAGCTCTGAGCAGATCAT-3’ | 214 | 55 |
|  | R:5’-GATCAGTTCCGGCACCTTG-3’ |  |  |
| Bcl-2 | F:5’-GGATCCAGGATAACGGAGGC-3’ | 150 | 55 |
|  | R: 5'-CCAGATAGGCACCCAGGGT-3' |  |  |
| Cyclin D1 | F: 5'-CCGAGAAGCTGTGCATCTAC-3' | 221 | 55 |
|  | R: 5'-CTTCACATCTGTGGCACAGAG-3' |  |  |
| Lin28b | F: 5'-AGCAAAGGTGGTGGAGAAGA-3' | 204 | 55 |
|  | R: 5'-TCTCGGTTTATCATGGAGATG-3' |  |  |

**Table S2** The sequences of control and UFC1 shRNAs

|  | Target sequence |
| --- | --- |
| sh-Ctrl | 5’-GATCCGTTCTCCGAACGTGTCACGTAATTCAAGAGATTACGTGACACGTTCGGAGAATTTTTTC-3’ |
| sh-UFC1-1 | 5’-CCGGAAGCACAGTGGTCTAAAAGTACTCGAGTACTTTTAGACCACTGTGCTTTTTTTG-3’ |
| sh-UFC1-2 | 5'-CCGGCTGTAGAAGGTTGAAGGGAAACTCGAGTTTCCCTTCAACCTTCTACAGTTTTTG-3' |
| sh-Lin28b | 5’-GATCCGGATATTCCAGTCGATGTATTCAAGAGATACATCGACTGGAATATCCTTTTTTC-3’ |

**Table S3 The association between UFC1 expression levels (–ΔCt) in tumor tissues and the clinicopathological features of gastric cancer patients**

| **Features** | **Number** | **UFC1** | | **Mean±SD** | **P value** |
| --- | --- | --- | --- | --- | --- |
|  |  | **High** | **Low** |  |  |
| **Gender** |  |  |  |  |  |
| Male | 57 | 38 | 19 | -2.37±2.65 | 0.528 |
| Female | 22 | 13 | 9 | -2.11±3.46 |  |
| **Age (years)** |  |  |  |  |  |
| ＜60 | 23 | 14 | 9 | -2.20±2.58 | 0.661 |
| ≥60 | 56 | 37 | 19 | -2.34±3.01 |  |
| **Tumor size(cm)** |  |  |  |  |  |
| ＜5 | 40 | 21 | 19 | -3.15±2.79 | 0.023 |
| ≥5 | 39 | 30 | 9 | -1.43±2.74 |  |
| **Differentiation^1^** |  |  |  |  |  |
| Moderate | 29 | 20 | 9 | -2.36±3.11 | 0.410 |
| Poor | 47 | 28 | 19 | -2.45±2.74 |  |
| **Lymphatic metastasis** |  |  |  |  |  |
| N0 | 24 | 6 | 18 | -3.04±2.06 | <0.001 |
| N1-3 | 55 | 45 | 10 | -1.98±3.13 |  |
| **Distal metastasis^1^** |  |  |  |  |  |
| M0 | 74 | 46 | 28 | -2.36±2.93 | 0.528 |
| M1 | 2 | 2 | 0 | 0.02±0.01 |  |
| **Venous or Perineural invasion** |  |  |  |  |  |
| Absent | 55 | 36 | 19 | -2.15±2.50 | 0.801 |
| Present | 24 | 15 | 9 | -2.65±3.64 |  |
| **Invasion depth** |  |  |  |  |  |
| T1 and T2 | 8 | 3 | 5 | -1.42±1.64 | 0.091 |
| T3 and T4 | 71 | 48 | 23 | -2.40±2.98 |  |
| **TNM stage^1^** |  |  |  |  |  |
| Ⅰ and Ⅱ | 26 | 9 | 17 | -2.81±2.12 | <0.001 |
| Ⅲ and Ⅳ | 50 | 39 | 11 | -2.03±3.25 |  |
| **Tumor location** |  |  |  |  |  |
| Antrum | 14 | 9 | 5 | -2.88±2.26 | 0.727 |
| Body | 7 | 4 | 3 | -2.63±2.68 |  |
| Angulus | 10 | 5 | 5 | -1.83±3.50 |  |
| Cardia | 28 | 18 | 10 | -1.79±2.68 |  |
| Others | 20 | 15 | 5 | -2.73±3.35 |  |

TNM tumor-node-metastasis.

^1^ indicates missing 3 cases.

**Table S4 The correlation between serum UFC1 expression levels (–ΔCt) and the clinicopathological characteristics of gastric cancer patients**

| **Features** | **Number** | **UFC1 expression** | | **Mean±SD** | **P value** |
| --- | --- | --- | --- | --- | --- |
|  |  | **High** | **Low** |  |  |
| **Gender** |  |  |  |  |  |
| Male | 46 | 32 | 14 | -3.35±2.46 | 0.894 |
| Female | 14 | 10 | 4 | -2.83±3.64 |  |
| **Age (years)** |  |  |  |  |  |
| ＜60 | 16 | 11 | 5 | -3.67±2.63 | 0.899 |
| ≥60 | 44 | 31 | 13 | -3.07±2.81 |  |
| **Tumor size(cm)** |  |  |  |  |  |
| ＜5 | 34 | 25 | 9 | -2.74±2.60 | 0.495 |
| ≥5 | 26 | 17 | 9 | -3.87±2.86 |  |
| **Differentiation^1^** |  |  |  |  |  |
| Moderate | 21 | 16 | 5 | -3.11±2.27 | 0.335 |
| Poor | 36 | 23 | 13 | -3.46±3.00 |  |
| **Lymphatic metastasis** |  |  |  |  |  |
| N0 | 17 | 8 | 9 | -3.89±3.08 | 0.015 |
| N1-3 | 43 | 34 | 9 | -2.97±2.60 |  |
| **Venous or Perineural invasion** |  |  |  |  |  |
| Absent | 44 | 32 | 12 | -3.18±2.69 | 0.756 |
| Present | 16 | 11 | 5 | -3.38±3.01 |  |
| **Invasion depth** |  |  |  |  |  |
| T1 and T2 | 4 | 2 | 2 | -4.07±1.81 | 0.366 |
| T3 and T4 | 56 | 40 | 16 | -3.17±2.81 |  |
| **TNM stage** |  |  |  |  |  |
| I and II | 19 | 9 | 10 | -4.20±2.63 | 0.009 |
| III and IV | 41 | 33 | 8 | -2.78±2.72 |  |

^1^ indicates missing 3 cases.

**Table S5 The correlation between exosomal UFC1 expression levels (–ΔCt) and the clinicopathological parameters of gastric cancer patients**

| **Features** | **Number** | **UFC1 expression** | | **Mean±SD** | **P value** |
| --- | --- | --- | --- | --- | --- |
|  |  | **High** | **Low** |  |  |
| **Gender** |  |  |  |  |  |
| Male | 46 | 32 | 14 | -2.90±2.21 | 0.728 |
| Female | 11 | 7 | 4 | -2.95±3.30 |  |
| **Age (years)** |  |  |  |  |  |
| ＜60 | 18 | 12 | 6 | -2.79±1.79 | 0.847 |
| ≥60 | 39 | 27 | 12 | -2.96±2.68 |  |
| **Tumor size(cm)** |  |  |  |  |  |
| ＜5 | 25 | 14 | 11 | -2.98±2.80 | 0.075 |
| ≥5 | 32 | 25 | 7 | -2.85±2.13 |  |
| **Differentiation^1^** |  |  |  |  |  |
| Moderate | 22 | 13 | 9 | -3.05±2.39 | 0.368 |
| Poor | 31 | 22 | 9 | -2.85±2.58 |  |
| **Lymphatic metastasis** |  |  |  |  |  |
| N0 | 14 | 3 | 11 | -4.81±2.35 | <0.001 |
| N1-3 | 43 | 36 | 7 | -2.29±2.12 |  |
| **Venous or Perineural invasion** |  |  |  |  |  |
| Absent | 42 | 26 | 16 | -3.11±2.32 | 0.109 |
| Present | 15 | 13 | 2 | -2.33±2.68 |  |
| **Invasion depth** |  |  |  |  |  |
| T1 and T2 | 4 | 1 | 3 | -5.22±2.97 | 0.088 |
| T3 and T4 | 53 | 38 | 15 | -2.73±2.32 |  |
| **TNM stage** |  |  |  |  |  |
| I and II | 16 | 3 | 13 | -4.64±2.32 | <0.001 |
| III and IV | 41 | 36 | 5 | -2.23±2.13 |  |

^1^ indicates missing 4 cases.
