# Supplementary material for: Long non-coding RNA UFC1 promotes gastric cancer progression by regulating miR-498/Lin28b
Source: J Exp Clin Cancer Res. 2018 Jul 3;37:134. doi: 10.1186/s13046-018-0803-6 (PMC6029056; doi:10.1186/s13046-018-0803-6)
Supplement: Supplementary file 2 — Figure S1. UFC1 knockdown inhibits gastric cancer cell proliferation, migration and invasion. Figure S2. UFC1 overexpression enhances gastric cancer cell proliferation, migration and invasion. Figure S3. Bioinformatic prediction of UFC1-binding miRNAs and target genes of miR-498. Figure S4. Relative expression levels of miR-498 and Lin-28b in gastric cancer cells and gastric cancer tissues. Figure S5. UFC1 overexpression antagonizes miR-498-medited inhibition of gastric cancer cell proliferation, migration and invasion. Figure S6. Lin28b knockdown inhibits gastric cancer cell proliferation, migration and invasion. Figure S7. Lin28b overexpression promotes gastric cancer cell proliferation, migration and invasion. Figure S8. UFC1 promotes gastric cancer cell proliferation, migration and invasion via the upregulation of Lin28b. (DOCX 19 kb) [file 13046_2018_803_MOESM2_ESM.docx]

**Figure legends**

**Figure S1. UFC1 knockdown inhibits gastric cancer cell proliferation, migration and invasion.** (**A**) The efficiency of UFC1 knockdown in GC cells was examined by qRT-PCR. (**B**) The growth of UFC1 knockdown GC cells was determined by using cell counting assay. (**C**) Cell colony formation assay for the proliferating ability of UFC1 knockdown GC cells. (**D**) The effects of UFC1 knockdown on the migration of GC cells were determined by using transwell migration assay. (**E**) Matrigel invasion assay was performed to determine the effects of UFC1 knockdown on GC cell invasion.

**Figure S2. UFC1 overexpression enhances gastric cancer cell proliferation, migration and invasion.** (**A**) The efficiency of UFC1 overexpression in GC cells was examined by qRT-PCR. (**B**) The growth of UFC1 overexpressing GC cells was determined by using cell counting assay. (**C**) Cell colony formation assay for the proliferating ability of UFC1 overexpressing GC cells. (**D**) The cell cycle distribution in UFC1overexpressing GC cells was determined by using flow cytometry. (**E**) The effects of UFC1 overexpression on the migration of GC cells were determined by using transwell migration assay. (**F**) Matrigel invasion assay was performed to determine the effects of UFC1 overexpression on GC cell invasion. (**G-H**) The effects of UFC1 overexpression on the expression of growth and metastasis-related genes (**G**) and proteins (**H**) in GC cells.

**Figure S3. Bioinformatic prediction of UFC1-binding miRNAs and target genes of miR-498.** (**A**) UFC1 sequence (ID: KJ809564.1) was retrieved from GenBank and submitted to miRDB website ([www.mirdb.org](http://www.mirdb.org)) for predicting the potential binding miRNAs. (**B**) The potential target genes of miR-498 were predicted by using online miRNA target prediction tools including Targetscan, DIANA, and miRDB. The binding sites in the 3’-UTR of Lin28b mRNA was shown (predicted by using Targetscan).

**Figure S4. Relative expression levels of miR-498 and Lin-28b in gastric cancer cells and gastric cancer tissues.** (**A**) The expression levels of miR-498 in gastric cancer tissues were determined by using qRT-PCR. (**B**) The expression levels of miR-498 in gastric cancer cells were determined by using qRT-PCR. (**C**) The expression levels of Lin28b in gastric cancer tissues were determined by using qRT-PCR. (**D**) The expression levels of Lin28b in gastric cancer cells were determined by using qRT-PCR.

**Figure S5. UFC1 overexpression antagonizes miR-498-medited inhibition of gastric cancer cell proliferation, migration and invasion.** (**A**) MGC-803 and BGC-823 cells were transfected with miR-498 in the presence or absence of UFC1. The growth of transfected cells was determined by using cell counting assay. (**B**) The cell cycle distribution of transfected cells was determined by using flow cytometry. (**C**) The percentage of apoptotic cells was determined by using flow cytometry. (**D**) The migration of transfected cells was determined by using transwell migration assay. (**E**) The invasion of transfected cells was determined by using matrigel invasion assay.

**Figure S6. Lin28b knockdown inhibits gastric cancer cell proliferation, migration and invasion** (**A**) Lin28b expression was silenced in gastric cancer cells by using shRNA. The efficiency of gene knockdown was validated by qRT-PCR. (**B**) The growth of sh-Lin28b transfected GC cells was determined by using cell counting assay. (**C**) Cell colony formation assays for the proliferating ability of sh-Lin28b transfected GC cells. (**D**) The cell cycle distribution in sh-Lin28b transfected GC cells was determined by using flow cytometry. (**E**) Flow cytometric analyses of cell apoptosis in sh-Lin28b transfected GC cells. (**F**) The effects of Lin28b knockdown on the migration of GC cells were determined by using transwell migration assay. (**G**) Matrigel invasion assay was performed to determine the effects of Lin28b knockdown on GC cell invasion. (**H-I**) The effects of LIn28b knockdown on the expression of growth and metastasis-related genes (**H**) and proteins (**I**) in GC cells.

**Figure S7. Lin28b overexpression promotes gastric cancer cell proliferation, migration and invasion.** (**A**) MKN-45 cells were transfected with Lin28b-overexpressing plasmid. The expression level of Lin28b in the transfected cells were determined by using qRT-PCR. (**B**) The growth of transfected cells was determined by using cell counting assay. (**C**) The proliferation of transfected cells was determined by using cell colony formation assay. (**D**) The migration and invasion of transfected cells was determined by using transwell migration assay and matrigel invasion assay. (**E**) The expression levels of proliferation- and EMT-related genes in the transfected cells were determined by using qRT-PCR.

**Figure S8. UFC1 promotes gastric cancer cell proliferation, migration and invasion via the upregulation of Lin28b.** MKN-45 cells were transfected with UFC1 in the presence or absence of Lin28b shRNA. (**A**) The growth of transfected cells was determined by using cell counting assay. (**B**) The proliferation of transfected cells was determined by using cell colony formation assay. (**C**) The migration and invasion of transfected cells was determined by using transwell migration assay and matrigel invasion assay.
